# Chirality-Regulated Spin-Polarization of Perovskite Nanoplates for Photocatalytic CO_2_ Reduction Reaction

**DOI:** 10.1021/jacs.5c11357

**Published:** 2025-08-19

**Authors:** Cheng-Chieh Lin, Shao-Ku Huang, Wei-Ni Tseng, Chun-Jen Su, Chao-Ching Huang, Chih-Ying Huang, Cheng-Yu Yu, Man-Hong Lai, Jia-Yuan Sun, Yu-Chiang Chao, Hua-Shu Hsu, Chih-Wei Luo, Yu-Ming Chang, Chia-Chun Chen, Chun-Wei Chen

**Affiliations:** † International Graduate Program of Molecular Science and Technology, 33561 National Taiwan University, Taipei 10617, Taiwan; ‡ Molecular Science and Technology Program, Taiwan International Graduate Program, Academia Sinica, Taipei 11529, Taiwan; § Department of Materials Science and Engineering, National Taiwan University, Taipei 10617, Taiwan; ∥ Department of Chemistry, 34879 National Taiwan Normal University, Taipei 11677, Taiwan; ⊥ National Synchrotron Radiation Research Center, Hsinchu 300092, Taiwan; # Center for Condensed Matter Sciences, National Taiwan University, Taipei 10617, Taiwan; ¶ Department of Electrophysics, 34914 National Yang Ming Chiao Tung University, Hsinchu 30010, Taiwan; ∇ Institute of Physics and Center for Emergent Functional Matter Science, National Yang Ming Chiao Tung University, Hsinchu 30010, Taiwan; ○ Department of Physics, National Taiwan Normal University, Taipei 11677, Taiwan; ◆ Department of Applied Physics, 63378 National Pingtung University, Pingtung 90044, Taiwan; †† Center of Atomic Initiative for New Materials, National Taiwan University, Taipei 10617, Taiwan

## Abstract

Manipulating spin polarizations of photoexcited electrons has been found to play a vital role in enhancing photocatalytic CO_2_ conversion by suppressing carrier recombination. In this work, photocatalytic CO_2_ reduction conversion efficiencies are significantly enhanced by chirality-regulated spin-polarization of CsPbBr_3_ perovskite nanocrystals. We propose the chirality-regulated perovskite thin films by incorporating chiral molecules (MBA:Br) into all-inorganic CsPbBr_3_ perovskite nanoplates (NPLs), resulting in (R)- and (S)-2D Ruddlesden–Popper perovskite (RPP)/NPL hybrids. In this configuration, the chiral 2D RPP perovskites offer a significant chiroptical response that promotes the generation of spin-polarized electrons. The chirality-regulated spin-polarization of 2D RPP/NPLs hybrid perovskite thin films has significantly suppressed charge carrier recombination rates, thereby enhancing the efficiency of photocatalytic CO_2_ reduction. By harnessing the synergistic effects of induced chirality and the application of an external magnetic field of 0.3 T, the photocatalytic CO_2_ reduction efficiencies of the chiral perovskites can be enhanced to be five times that of the pristine CsPbBr_3_ perovskite NPLs. The interplay between structure, chirality, spin polarization, and carrier dynamics associated with the enhanced photocatalytic activity of perovskite nanocrystals was systematically analyzed using grazing-incidence wide-angle X-ray scattering (GIWAXS) spectroscopy, magnetic circular dichroism (MCD) spectroscopy, and time-resolved photoluminescence (PL) techniques. Our results pave the way for the manipulation of spin-polarized electrons through chirality-regulated perovskite nanocrystals, significantly enhancing photocatalytic CO_2_ reduction efficiencies and highlighting their strong potential for future solar-to-fuel conversion applications.

## Graphical abstract

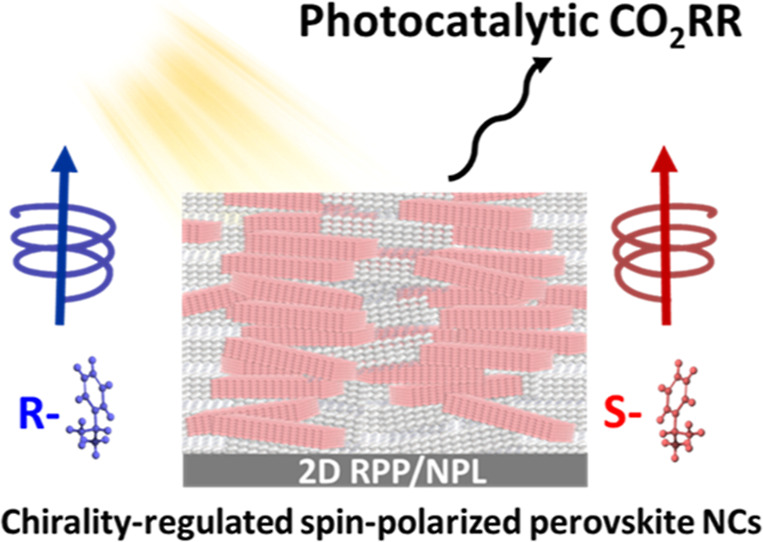

## Introduction

Artificial photosynthesis, which employs solar-driven CO_2_ reduction to produce high-value fuels, has recently garnered significant attention, offering a promising solution to the challenges of climate change and the energy crisis.^1–3^ Photocatalytic CO_2_ reduction has emerged as a promising method to convert CO_2_ into valuable fuels and chemicals using renewable energy sources. However, due to the multielectron transfer involved in photocatalytic CO_2_ reduction, the efficiency of solar-driven CO_2_ conversion remains challenging for practical industrial applications.^4,5^ Several critical factors impact the conversion efficiency of photocatalytic CO_2_ reduction, such as visible-light absorption, CO_2_ adsorption, charge separation, carrier transport, and redox reactions.^2,3,6^ Significant research efforts have focused on optimizing these factors to develop highly efficient photocatalysts for CO_2_ reduction.^2,3,6,7^ In particular, the control of photogenerated carrier dynamics in photocatalysts—such as exciton dissociation, charge separation, and carrier recombination—has become essential for promoting photocatalytic performance in CO_2_ reduction.^8–10^ Recently, the manipulation of spin-polarized electrons in photocatalysts has emerged as an effective strategy to enhance photocatalytic CO_2_ reduction efficiencies.^11–13^ Because photocatalysis usually requires a rapid charge transfer and a prolonged lifetime of intermediate species for redox reactions, the spin polarizations of photoexcited electrons are found to play a vital role in the suppression of carrier recombination.^14^ The polarization of spin states in photocatalysts can be achieved by doping the magnetic elements,^12,13,15^ creating defects or vacancies,^11,16–18^ and can be further synergistically enhanced by applying an external magnetic field.^19–21^

Polarizing the spin state of the photoinduced charges by chiral molecules could be another effective strategy to manipulate the spin-polarized electrons of photocatalysts. Chiral ligands attached to the surface of halide perovskites recently led to the observation of intriguing chiral photonics resulting from spin-polarized optical absorption and emission through the chiral transfer of molecules.^22,23^ These noncentrosymmetric chiral perovskites have gained tremendous research attention in the chiral photonic community.^24–27^ In this work, we would like to demonstrate that photocatalytic CO_2_ reduction conversion efficiencies can be significantly enhanced by chirality-regulated spin-polarization of CsPbBr_3_ perovskite nanocrystals. The chirality-regulated spin-polarization of halide perovskite nanocrystals significantly suppressed charge carrier recombination rates, thereby enhancing the efficiency of photocatalytic CO_2_ reduction. By harnessing the synergistic effects of induced chirality and the application of an external magnetic field of 0.3 T, the photocatalytic CO_2_ reduction efficiencies of the chiral perovskites can be enhanced to be five times that of the pristine CsPbBr_3_ perovskite NPLs. The interplay between structure, chirality, spin-polarization, and the photocatalytic activity of perovskite nanocrystals was systematically analyzed by grazing-incidence wide-angle X-ray scattering (GIWAXS) spectroscopy, magnetic circular dichroism (MCD) spectroscopy, and time-resolved PL techniques. Our result shows that manipulating spin-polarized electrons in chiral-regulated semiconductors offers an effective strategy to boost photocatalytic CO_2_ reduction efficiencies.

## Results and Discussion

The recent success in incorporating chiral organic molecules into 2D organic–inorganic hybrid perovskites has drawn considerable research interest in the field of chiroptics.^22,23^ For example, 2D Ruddlesden–Popper perovskite (RPP) chiral perovskites are composed of varying numbers of self-assembled inorganic layers separated by chiral organic ligands. These materials exhibit remarkable chirality-induced optical properties such as circular dichroism (CD) and circularly polarized luminescence (CPL) due to spin-polarized optical absorption and emission via the chiral transfer of molecules.^22,23^ Inspired by the strong chiroptical response of 2D RPP chiral perovskites, we propose designing materials by synthesizing the 2D RPP/nanoplate (NPL) hybrid perovskite structure as illustrated in the schematic diagram in Figure 1a. This strategy aims to facilitate chirality-regulated spin-polarized photocatalysis for CO_2_ reduction, incorporating 2D RPP chiral perovskite nanocrystals into the all-inorganic perovskite matrix of CsPbBr_3_ NPLs. First, we synthesize CsPbBr_3_ perovskite NPLs as building blocks for the fabrication of 2D RPP/NPL hybrid perovskite thin films. The pristine CsPbBr_3_ NPLs were synthesized using the traditional hot injection method, as detailed in the Supporting Information.^28,29^

**Figure 1:**
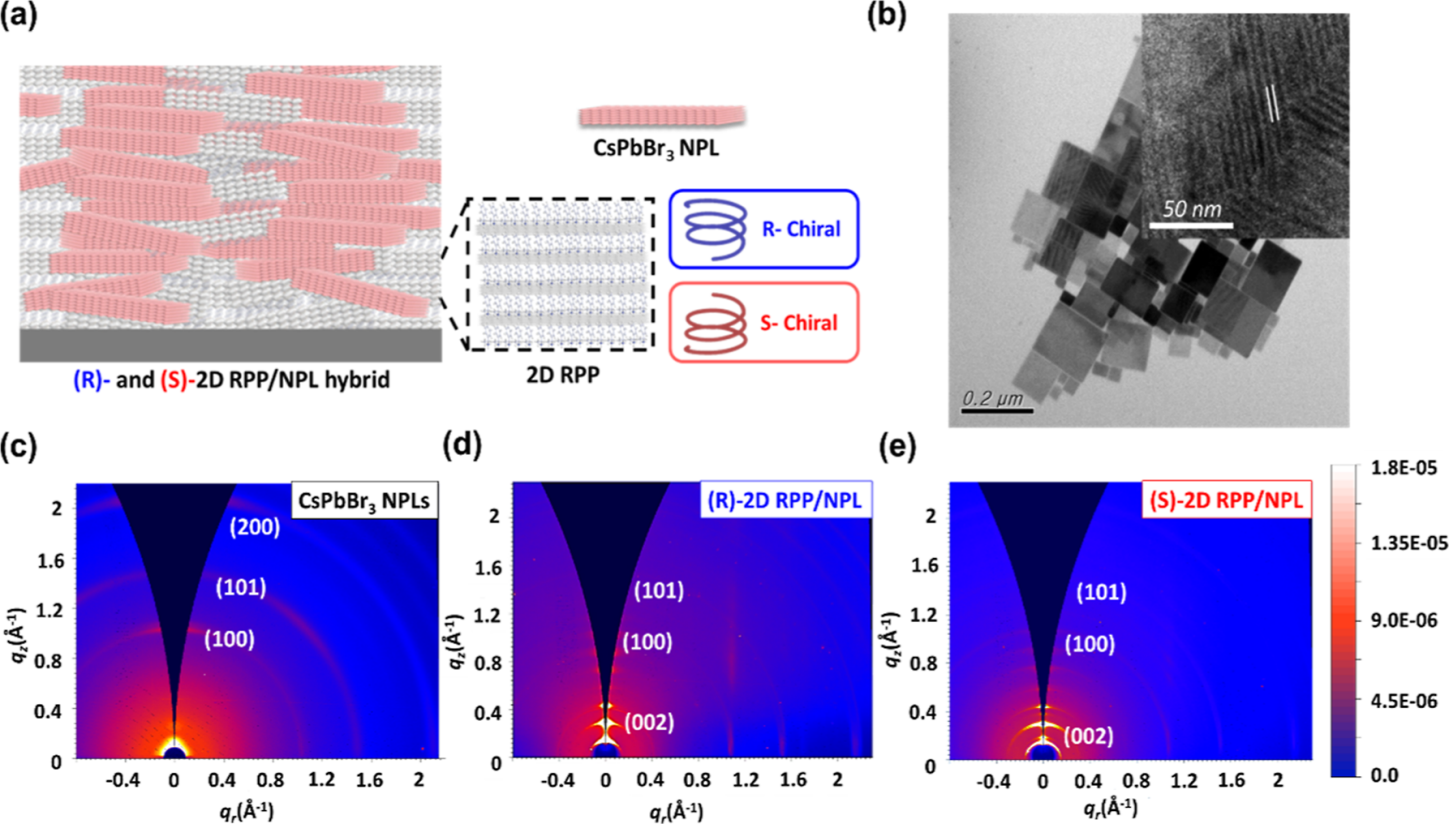
Morphology and structural characterization of CsPbBr_3_ perovskite NPLs, (R)-2D RPP/NPL, and (S)-2D RPP/NPL, respectively. (a) Schematic diagram of the (R)- and (S)-2D RPP/NPL hybrid perovskite thin films. (b) Morphology of CsPbBr_3_ NPLs as observed in the TEM image. The HR-TEM image of the cross-sectional CsPbBr_3_ NPLs in the inset. The 2D GIWAXS patterns of (c) CsPbBr_3_ NPLs, (d) (R)-2D RPP/NPL and (e) (S)-2D RPP/NPL.

The transmission electron microscopy (TEM) image in Figure 1b reveals that the CsPbBr_3_ perovskite NPLs exhibit a rectangular morphology, featuring lateral dimensions of approximately 100–200 nm. The inset in Figure 1b displays a cross-sectional image of the CsPbBr_3_ perovskite NPLs, obtained using high-resolution transmission electron microscopy (HR-TEM), revealing a thickness of approximately ∼3 nm. This observation is consistent with the atomic force microscopy (AFM) height profile shown in the Supporting Information. Here, we propose a two-step synthesis protocol to fabricate 2D RPP/NPL hybrid perovskite thin films, by employing chiral R/S-methylbenzylammonium bromide (chiral R/S-MBA:Br) molecules as organic ligands in conjunction with a self-recrystallization process of CsPbBr_3_ perovskite NPLs.^22^ The details of the two-step synthesis processes of the 2D hybrid RPP/NPL perovskite thin films are described in the Supporting Information.^30,31^ In brief, the chiral MBA:Br molecules are initially deposited onto a substrate, while concurrently heating the substrate to 70 °C to evaporate the majority of the residual solvent, resulting in the formation of uniform chiral molecular thin films. Recent studies suggest that perovskite nanocrystal structures are susceptible to phase transformation due to environmental factors such as surfactants, solvents, composition, temperature, and pressure.^32,33^ These transformations can lead to structural cracks and the formation of nucleation sites, resulting in the phase transformation during the recrystallization.^30,31^ Consequently, the second step in our two-step synthesis protocol is the coating of CsPbBr_3_ perovskite NPLs on the precoated chiral molecular films. When the CsPbBr_3_ perovskite NPLs come into contact with the chiral MBA:Br molecular films, they might undergo recrystallization due to environmental changes. This interaction results in the occurrence of structural cracking, nucleation, and further recrystallization in the perovskite due to its reaction with the chiral MBA/Br molecules,^22^ leading to the formation of 2D RPP/NPL hybrid perovskite thin films.

To further investigate the structural compositions of 2D RPP/NPL hybrid perovskite thin films, two-dimensional grazing-incidence wide-angle X-ray scattering (2D-GIWAXS) was employed (at the 23A SWAXS beamline of Taiwan Light Source (TLS) at the National Synchrotron Radiation Research Center (NSRRC)) to characterize the crystallinity and lattice orientations of the synthesized 2D RPP/NPL hybrid perovskite thin films.^22,34–36^ Figure 1c presents the GIWAXS scattering pattern for the pristine CsPbBr_3_ perovskite NPLs, where the observed scattering peaks corresponding to the (100), (101), and (200) planes are the typical characteristics of the three-dimensional (3D) perovskite crystal lattice structure.^34,37–39^ The result suggests that the CsPbBr_3_ perovskite nanoplates (NPLs) have a crystal structure similar to that of 3D CsPbBr_3_ perovskites^40,41^ and exhibit an anisotropic, layered morphology with an ultrathin thickness of only a few nanometers. By contrast, the GIWAXS patterns of R- and S-2D RPP/NPL hybrid CsPbBr_3_ perovskite thin films, as shown in Figure 1d,e, respectively, exhibit low scattering wavevector signals at *q* < 1 Å^–1^. The low scattering wavevectors observed for 2D RPP/NPL hybrid perovskite thin films indicate the presence of a 2D organic–inorganic hybrid RPP crystal structure. The (002) scattering peak of the 2D RPP at *q* = 0.16 Å^–1^ results from the interlayer spacing of about 3.9 nm, corresponding to the crystal structure of (MBA)_2_Cs_3_Pb_4_Br_13_ perovskite with *n* = 4.^42^ By subtracting the thickness of organic chiral ligands, the inorganic perovskite layers have a thickness of ∼3 nm, which is consistent with the result obtained by the cross-sectional HRTEM image of the CsPbBr_3_ perovskite NPLs as shown in the inset of Figure 1b. Furthermore, there are 3D characteristics found at 1.08 Å^–1^ and 1.50 Å^–1^ in these two samples of 2D RPP/NPL hybrid perovskite thin films. The results indicated that the formation of (R)- and (S)-2D RPP/NPL hybrid perovskite thin films can be achieved using our two-step synthesis protocol. Additional X-ray diffraction (XRD) measurements, provided in the Supporting Information, further confirm the 2D/3D hybrid nature of these films. In this hybrid structure, the 2D RPP chiral perovskites, which are embedded within the nonchiral perovskite NPL matrix, play a key role in generating spin-polarized electrons through chirality transfer. Nevertheless, the (R)-2D RPP/NPL hybrid perovskite thin film seems to have higher crystallinity and orientation than its (S)-2D RPP/NPL counterpart. The (002) crystallographic orientation of the (S)-2D RPP/NPL hybrid reveals a distinct arc of diffracted intensity with a larger azimuthal angle, resulting from its less oriented film structure when compared to the (R)-2D RPP/NPL hybrid perovskite thin film.^43^ The exact reasons for the differences in crystallinity and orientation between these two samples, produced using our two-step synthesis process, remain unclear at this stage. Possible explanations may involve the asymmetry of the chiral molecules and their ability to establish different intermolecular interactions with the crystal surface, potentially affecting the growth crystallinity and chiroptical properties of the materials.^44^ More detailed investigations into this issue of affecting the growth crystallinity and the corresponding chiroptical properties of materials are currently in progress.

The linear absorption and photoluminescence spectra for the pristine CsPbBr_3_ NPLs, (R)- and (S)-2D RPP/NPL hybrid perovskites are presented in Figure 2a. Typically, the 2D RPPs possess a naturally formed “quantum-well (QW)-like” structure, comprising inorganic perovskite layers made up of corner-sharing PbX_6_ octahedra that are positioned between organic spacers. The parameter (*n*) indicates the number of inorganic perovskite layers per unit cell, which determines the width of the quantum well and subsequently affects the band gaps and optical properties of the 2D RPPs.^43,44^ Both the excitonic absorption and PL emission peaks from the pristine CsPbBr_3_ NPLs and (R)- and (S)-2D RPP/NPL hybrid perovskite thin films exhibit a blue shift compared to the typical absorption and emission peaks of the conventional CsPbBr_3_ nanocrystals with a larger size (>10 nm),^28^ arising from the quantum confinement effect. The excitonic absorption peaks and PL emission peaks for the pristine CsPbBr_3_ NPLs and the (R)- and (S)-2D RPP/NPL hybrid perovskite thin films are found at similar positions, with slightly broader spectral profiles of the 2D RPP/NPL hybrid films compared to their pristine CsPbBr_3_ NPL counterpart. This suggests that the quantum well width and band gap of the 2D RPPs in the (R)- and (S)-2D RPP/NPL hybrid perovskite thin films are similar to those of the pristine CsPbBr_3_ NPLs. Compared with the previous report on the optical absorption and PL emission peak data,^45^ the thickness of the CsPbBr_3_ NPLs and also the number of inorganic perovskite layers per unit cell in the 2D RPP are estimated to be *n* = 4, consistent with the above GIWAXS analyses.

**Figure 2:**
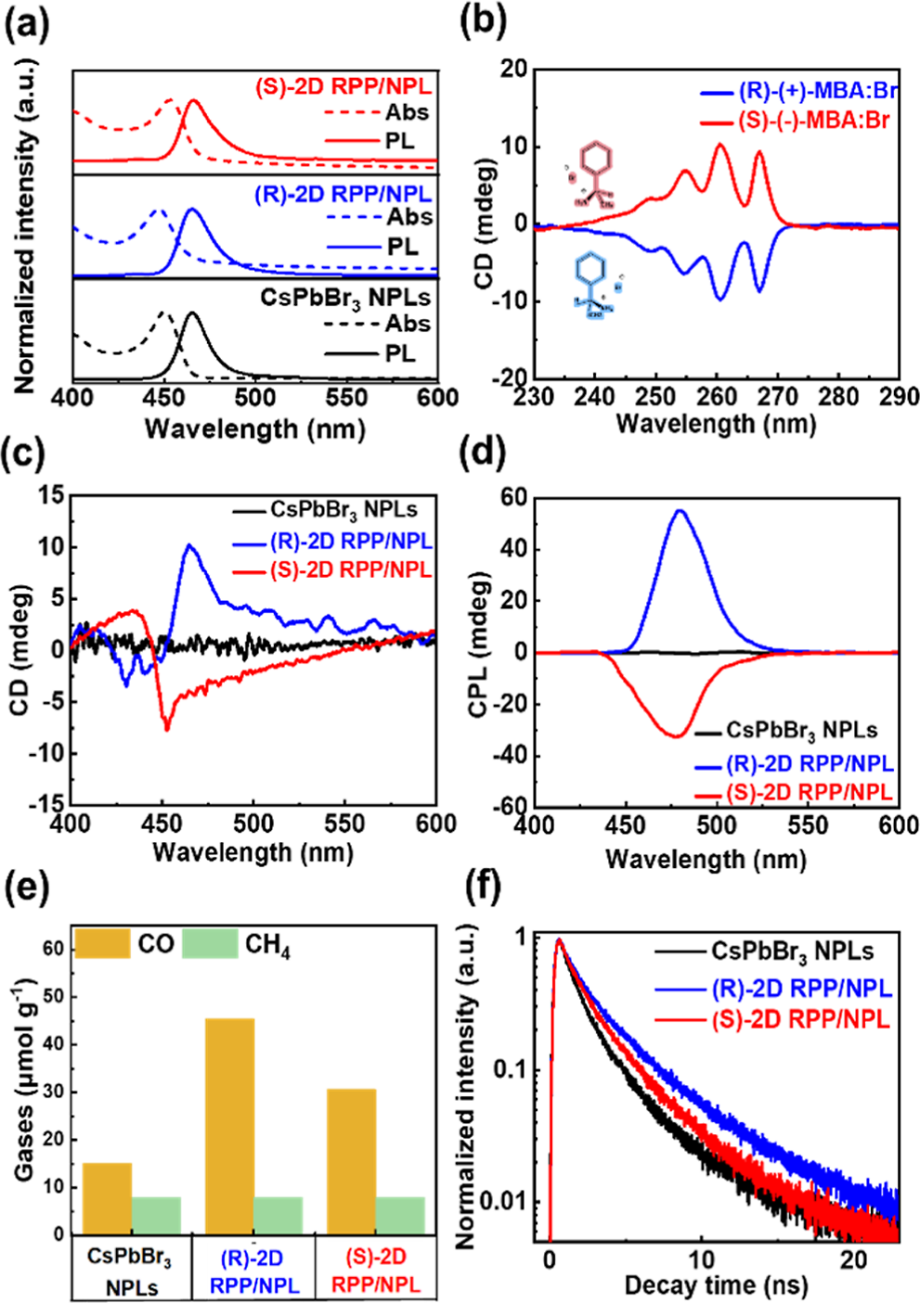
Optical properties, circularly polarized properties, and photocatalytic performance of CsPbBr_3_ NPLs, (R)-2D RPP/NPL, and (S)-2D RPP/NPL, respectively. (a) The PL and UV–vis absorption spectra (Abs) of CsPbBr_3_ NPLs, (R)-2D RPP/NPL, and (S)-2D RPP/NPL. (b) The CD spectra of the chiral molecules (R)-(+)-MBA:Br and (S)-(−)-MBA:Br. (c) The CD spectra of CsPbBr_3_ NPLs, (R)-2D RPP/NPL, and (S)-2D RPP/NPL, prepared from the two-step synthesis process. (d) The CPL spectra of CsPbBr_3_ NPLs, (R)-2D RPP/NPL, and (S)-2D RPP/NPL. (e) The photocatalytic performance of CsPbBr_3_ NPLs, (R)-2D RPP/NPL, and (S)-2D RPP/NPL were measured in the product yields of CO and CH_4_ gases over 6 h under light irradiation. (f) The TRPL of CsPbBr_3_ NPLs, (R)-2D RPP/NPL, and (S)-2D RPP/NPL.

Although the linear absorption and photoluminescence spectra of the pristine CsPbBr_3_ NPLs, (R)- and (S)-2D RPP/NPL hybrid perovskites are similar, they show significant discrepancy in the chiroptic response. First of all, CD spectroscopy was employed to confirm the chirality characteristic of R-(+)-MBA:Br and S-(−)-MBA:Br bromide salt. Figure 2b shows that the two chiral molecules exhibit opposite CD absorption features, demonstrating their ability to absorb spin-polarized light of opposite handedness preferentially, which is known as the Cotton effect.^46,47^ While the CD signals of the chiral molecules themselves do not extend beyond 280 nm, the 2D RPP/NPL hybrid films consisting of R-(+)-MBA:Br and S-(−)-MBA:Br chiral molecules exhibit pronounced CD spectra in the 400–500 nm visible light region, as shown in Figure 2c. For the (R)- and (S)-2D RPP/NPL hybrid perovskite thin films, the CD spectra demonstrate distinct dispersive features centered at the excitonic absorption of perovskite NPLs, indicating chirality transfer from the organic chiral molecules to the CsPbBr_3_ perovskite nanocrystals. This observation of chirality transfer can be explained by recent theoretical calculations on chirality transfer in 2D chiral perovskite.^48^ The simulation results suggest that the CD response primarily arises from transferred chirality due to structural asymmetry, particularly out-of-plane distortions and helicity within the inorganic metal-halide layers, rather than directly from the chiral organic cations. By contrast, the pristine CsPbBr_3_ NPLs thin film without the incorporation of chiral molecules exhibits no detectable CD response. Figure 2d exhibits the CPL spectra of the CsPbBr_3_ NPLs, (R)- and (S)-2D RPP/NPL hybrid perovskite thin films. There is no CPL signature for the pristine CsPbBr_3_ NPL thin film, while pronounced and opposite CPL characteristics peaked at peaking at ∼470 nm for both (R)- and (S)-2D RPP/NPL hybrid perovskite thin films can be observed. The CPL signatures closely align with the CD absorption features near the band-edge absorption at 460 nm. These findings highlight the distinct CD and CPL characteristics of the (R)- and (S)-2D RPP/NPL hybrid perovskite thin films. They confirm the effective transfer of chirality from the chiral molecules to the 2D RPP/NPL hybrid perovskite thin films, achieved through the incorporation of chiral molecules in accordance with our two-step synthesis protocol. We also used the racemic methylbenzylammonium bromide (*rac*-MBA:Br) to prepare the achiral perovskite as a control sample. There are no detectable signals for both CD and CPL spectra of the *rac*-2D RPP/NPL hybrid perovskite thin film as shown in the Supporting Information. Additionally, the CD and CPL intensities of the (R)-2D RPP/NPL hybrid perovskite thin film appear to be stronger than those of the (S)-2D RPP/NPL hybrid perovskite counterpart. This difference could be attributed to the higher crystallinity and orientation of the (R)-2D RPP/NPL hybrid CsPbBr_3_ thin film, as revealed by the above GIWAXS spectra in Figure 2d,e.

Next, we examine the effect of enhanced spin-polarized electrons on the photocatalytic CO_2_RR of the pristine CsPbBr_3_ NPL, (R)- and (S)-2D RPP/NPL hybrid perovskite thin films. The photocatalytic CO_2_RR performance was assessed by subjecting the materials to CO_2_-saturated water vapor under 6 h of simulated solar illumination (AM 1.5G, 100 mW/cm^2^). Two control experiments (in the dark and in a N_2_ atmosphere under illumination) and ^13^CO_2_ isotopic labeling experiments were also performed. The results demonstrate that the products generated from photocatalytic CO_2_RR over the pristine CsPbBr_3_ NPL, (R)- and (S)-2D RPP/NPL hybrid perovskite thin films originated from CO_2_ rather than surface ligands, as shown in Figures S6 and S7. The primary products generated during the photocatalytic CO_2_RR were CO and CH_4_, as shown in Figure 2e. There was no significant variation in the production of CH_4_ (∼8 μmol g^–1^) for all three samples. By contrast, the (R)- and (S)-2D RPP/NPL hybrid perovskite thin films exhibit significantly higher product yields in the production of CO compared to the CsPbBr_3_ NPLs thin film. The CO production yields for the CsPbBr_3_ NPLs, (R)- and (S)-2D RPP/NPL hybrid thin films are 15.1, 45.5, and 30.6 μmol g^–1^. The improved photocatalytic CO_2_ reduction performance in (R)- and (S)-2D RPP/NPL hybrid perovskite thin films compared to pristine CsPbBr_3_ NPLs, is primarily attributed to the increased generation of spin-polarized electrons resulting from the chirality transfer of the incorporated chiral molecules. To explore the charge carrier dynamics in CsPbBr_3_ NPLs, (R)- and (S)-2D RPP/NPL hybrid perovskite thin films, TRPL measurements were performed to assess the PL carrier lifetimes of the respective samples, as shown in Figure 2f. The average carrier lifetime of CsPbBr_3_ NPLs was measured at τ_NPL_ = 2.82 ns, significantly shorter than the extended lifetimes observed for (R)-2D RPP/NPL (τ_R_ = 4.07 ns) and (S)-2D RPP/NPL (τ_S_ = 3.49 ns) perovskite hybrids. The incorporation of chiral molecules into the (R)- and (S)-2D RPP/NPL hybrid perovskite thin films results in the formation of enhanced spin-polarized bands, as evidenced by the distinct CD signals. As a result, the photoexcited spin-polarized electrons in the conduction band (CB) of these chiral-regulated perovskites are likely to undergo spin relaxation (or spin-flip) and lose their original spin direction during the charge-transfer process, attributed to the strong spin–orbital coupling present in the material.^16,49^ Accordingly, the carrier recombination rate is largely suppressed because of the lack of spin-polarized holes with the same spin direction in the valence band (VB), leading to a longer carrier recombination lifetime as revealed from TRPL spectra. The prolonged carrier recombination lifetime, attributed to the presence of these spin-polarized bands induced by chirality transfer in the (R)- and (S)-2D RPP/NPL hybrid perovskite thin films, contributes to the improved photocatalytic CO_2_ reduction efficiencies compared to pristine CsPbBr_3_ NPLs. Additionally, the higher production yield and longer carrier lifetime observed in the (R)-2D RPP/NPL hybrid perovskite thin film, in comparison to the (S)-2D RPP/NPL counterpart, can primarily be ascribed to the higher crystallinity and orientation of the (R)-2D RPP/NPL hybrid perovskite thin film, as mentioned above. The improved photocatalytic CO_2_ reduction performance in (R)- and (S)-2D RPP/NPL hybrid perovskite thin films compared to pristine CsPbBr_3_ NPLs, is primarily attributed to the increased generation of spin-polarized electrons resulting from the chirality transfer of the incorporated chiral molecules. For comparison, we also performed the control experiment of the photocatalytic CO_2_ reduction performance from the achiral *rac*-2D RPP/NPL hybrid perovskite thin film. The rac-2D RPP/NPL film exhibited a CO production yield of 15.3 μmol g^–1^, similar to that of pristine CsPbBr_3_ NPLs. These results highlight the critical role of chirality-regulated spin-polarization in enhancing photocatalytic CO_2_ reduction performance in the chiral 2D RPP/NPL hybrid films. The use of chirality-regulated perovskite nanocrystals promotes the generation of spin-polarized electrons, which in turn prolong carrier recombination lifetimes and enhance the efficiency of photocatalytic CO_2_ reduction. The chirality-regulated spin polarization approach for photocatalytic CO_2_ reduction offers several unique advantages. Notably, it eliminates the need for magnetic doping^12^ or the introduction of defects and vacancies,^11^ which often lead to structural imperfections and trap states that may hinder charge transport. Additionally, it provides molecular tunability and versatility—chiral molecules can be chemically tailored via modifications to their backbone, functional groups, or handedness (enantiomers), enabling fine control over spin polarization strength and interfacial interactions. These features make chirality-regulated spin polarization a promising strategy for enhancing the efficiency of photocatalytic CO_2_ reduction.

Moreover, the spin polarization of electrons in semiconductors can be further enhanced by applying an external magnetic field, corresponding to the effect of Zeeman splitting.^50,51^ Figure 3a,b display the MCD spectroscopy of (R)- and (S)-2D RPP/NPL hybrid perovskite thin films under a magnetic field of 0 and 0.3 T, respectively. The presence of MCD signals in the (R)- and (S)-2D RPP/NPL hybrid perovskite thin films, which exhibit increased intensities under an external magnetic field (0.3 T) compared to the original CD signals (0 T), indicates the enhancement of spin-polarized bands due to Zeeman splitting.^50,51^ The result suggests that the spin polarization of the photoexcited carriers of (R)- and (S)-2D RPP/NPL hybrid perovskite thin films can be further enhanced under an external magnetic field. Next, the photocatalytic CO_2_RR performances of pristine CsPbBr_3_ NPLs, (R)- and (S)-2D RPP/NPL hybrid perovskite thin films with and without an external magnetic field were measured as shown in Figure 3c. Here, a permanent magnet, which does not need an external power supply, serves as the source of the magnetic field. The experimental setup for the photocatalytic CO_2_RR using a permanent magnet is depicted in Figure S3. An external magnetic field of 0.3 T is applied to enhance the photocatalytic performance of the chirality-regulated perovskites. Both CO and CH_4_ remain the primary products of the photocatalytic CO_2_RR for all three samples under the magnetic field. There is only a slight variation in the production of CH_4_ for all three samples, regardless of the presence of a magnetic field. For the pristine CsPbBr_3_ NPLs, the CO product yields are nearly unchanged under an external magnetic field, recorded at 15.1 μmol g^–1^ at 0 T and 16.6 μmol g^–1^ at 0.3 T. By contrast, the (R)- and (S)-2D RPP/NPL hybrid perovskite thin films exhibit a substantial increase in CO production, with yields rising from 45.5 μmol g^–1^ at 0 T to 75.3 μmol g^–1^ at 0.3 T, and from 30.6 μmol g^–1^ at 0 T to 49.3 μmol g^–1^ at 0.3 T, respectively, over a 6 h duration. The improvement in photocatalytic CO_2_ conversion efficiency exceeds 60% for the photocatalytic performance of the chirality-regulated perovskites under a magnetic field of 0.3 T. The enhanced photocatalytic CO_2_RR in the (R)- and (S)-2D RPP/NPL hybrid perovskite thin films under an external magnetic field is mainly attributed to the enhanced spin-polarized electrons, as revealed by the above MCD spectra. The result represents approximately a 5-fold enhancement for the (R)-2D RPP/NPL hybrid and a 3-fold enhancement for the (S)-2D RPP/NPL hybrid when subjected to an external magnetic field of 0.3 T, compared to the pristine CsPbBr_3_ NPLs. Figure 3d exhibits the superior photocatalytic stability and reproducibility of both pristine CsPbBr_3_ NPLs and (R)-2D RPP/NPL hybrid perovskite thin films, showing consistent product yields of CO and CH_4_ after five consecutive 30 h cycles under a 0.3 T external magnetic field. This result indicates the excellent stability of photocatalytic performance achieved by chirality-regulated perovskite nanocrystals.

**Figure 3:**
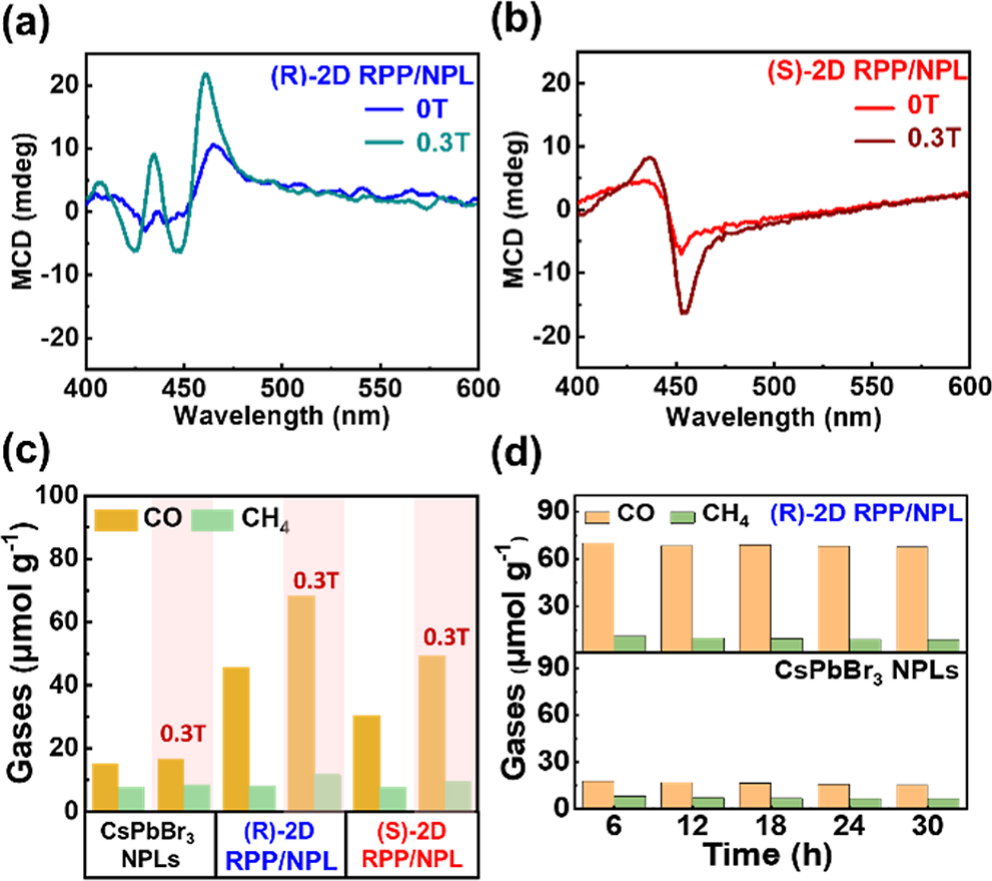
MCD spectra and photocatalytic performance of CsPbBr_3_ NPLs, (R)-2D RPP/NPL, and (S)-2D RPP/NPL with and without an external magnetic field (0.3 T). The MCD spectra of (a) (R)-2D RPP/NPL and (b) (S)-2D RPP/NPL, both with and without an external magnetic field (0 and 0.3 T). (c) Photocatalytic yield measurements of CsPbBr_3_ NPLs, (R)-2D RPP/NPL, and (S)-2D RPP/NPL with and without an external magnetic field (0 and 0.3 T) over 6 h of irradiation. (d) Photocatalytic stability comparison of pristine CsPbBr_3_ NPLs and R-2D RPP/NPL. Both samples show excellent stability over a 30 h reaction period under a 0.3 T external magnetic field.

The corresponding carrier dynamics of pristine CsPbBr_3_ NPLs and the (R)- and (S)-2D RPP/NPL hybrid perovskite thin films, both with and without an external magnetic field, were further investigated using TRPL measurements, as illustrated in Figure 4a–c. The fitting model and the corresponding components of TRPL decay curves of these samples are shown in the Supporting Information. The average carrier lifetime of the pristine CsPbBr_3_ NPLs remains nearly constant, shifting only slightly from 2.82 to 2.85 ns when the external magnetic field is increased from 0 to 0.3 T. (Figure 4a) By contrast, the (R)- and (S)-2D RPP/NPL hybrid perovskite thin films exhibit enhanced carrier lifetimes, increasing from 4.07 to 6.02 ns (Figure 4b) and from 3.49 to 4.61 ns (Figure 4c), respectively, under a 0.3 T magnetic field. Under an external magnetic field, (R)- and (S)-2D RPP/NPL hybrid perovskite thin films exhibit enhanced electron spin polarization, leading to reduced carrier recombination and prolonged lifetimes of photogenerated charge carriers.

**Figure 4:**
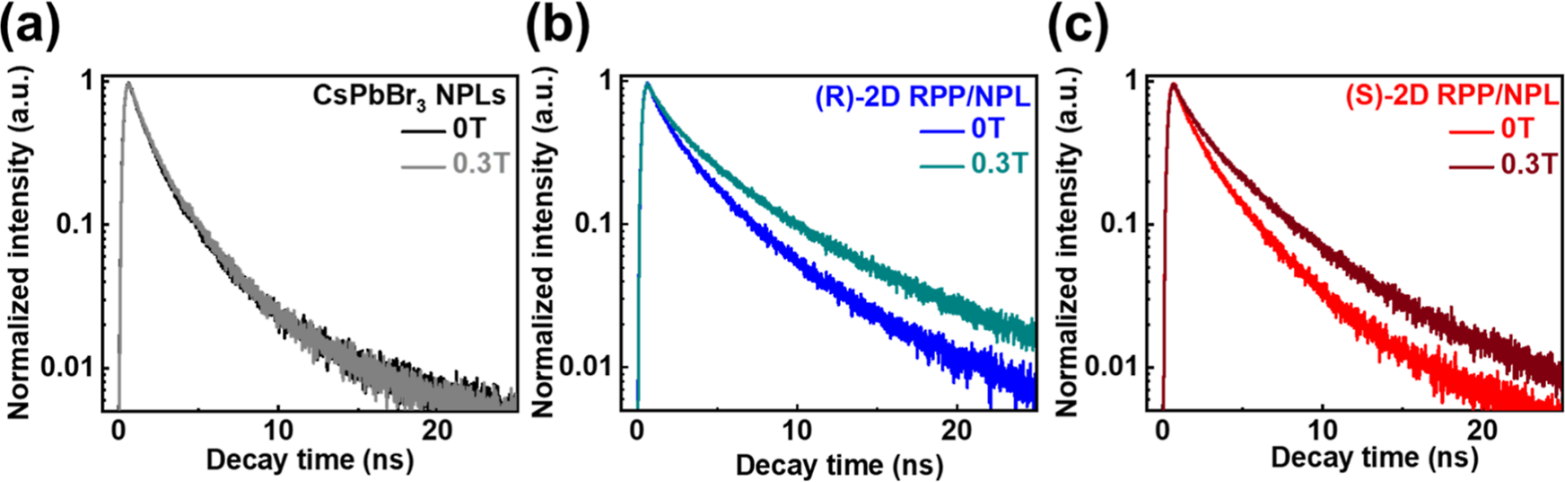
Carrier dynamics of CsPbBr_3_ NPLs, (R)-2D RPP/NPL, and (S)-2D RPP/NPL with and without an external magnetic field (0.3 T). The TRPL spectra of (a) CsPbBr_3_ NPLs, (b) (R)-2D RPP/NPL, and (c) (S)-2D RPP/NPL with and without an external magnetic field (0.3 T).

Figure 5 depicts the relationship between spin polarization manipulation in chirality-regulated 2D RPP/NPL hybrid perovskite thin films and the enhancement of photocatalytic CO_2_ reduction reaction (CO_2_RR) performance. In both (R)- and (S)-2D RPP/NPL hybrids, spin-polarized band structures are formed through the effective transfer of chirality from the chiral molecules to the perovskite NPLs, resulting in the formation of asymmetric DOS for spin-up and spin-down electrons. By contrast, the pristine CsPbBr3 NPLs with symmetric DOS lack spin-polarized electrons. Under light illumination, photoexcited spin-polarized electrons in the conduction band (CB) of chirality-regulated perovskite NPLs are generally susceptible to spin relaxation or spin-flip transitions during charge transfer, due to strong spin–orbit coupling and hyperfine interactions.^12,16,49^ The carrier recombination rate is significantly suppressed, as spin-polarized holes with matching spin orientation in the valence band (VB) are limited.^12^ This effect may contribute to the extended carrier lifetimes observed in both (R)- and (S)-2D RPP/NPL hybrids, where the suppressed carrier recombination contributes to the enhanced photocatalytic CO_2_RR performance of the chiral hybrid systems. Additionally, applying an external magnetic field induces Zeeman splitting in both (R)- and (S)-2D RPP/NPL hybrids, further separating spin-polarized bands and increasing the population of spin-polarized charge carriers, thereby resulting in a longer carrier lifetime and enhancing overall performance. The above mechanism highlights the correlation between spin polarization control in chirality-regulated 2D RPP/NPL hybrid perovskite thin films and the enhanced performance of photocatalytic CO_2_ reduction (CO_2_RR).

**Figure 5:**
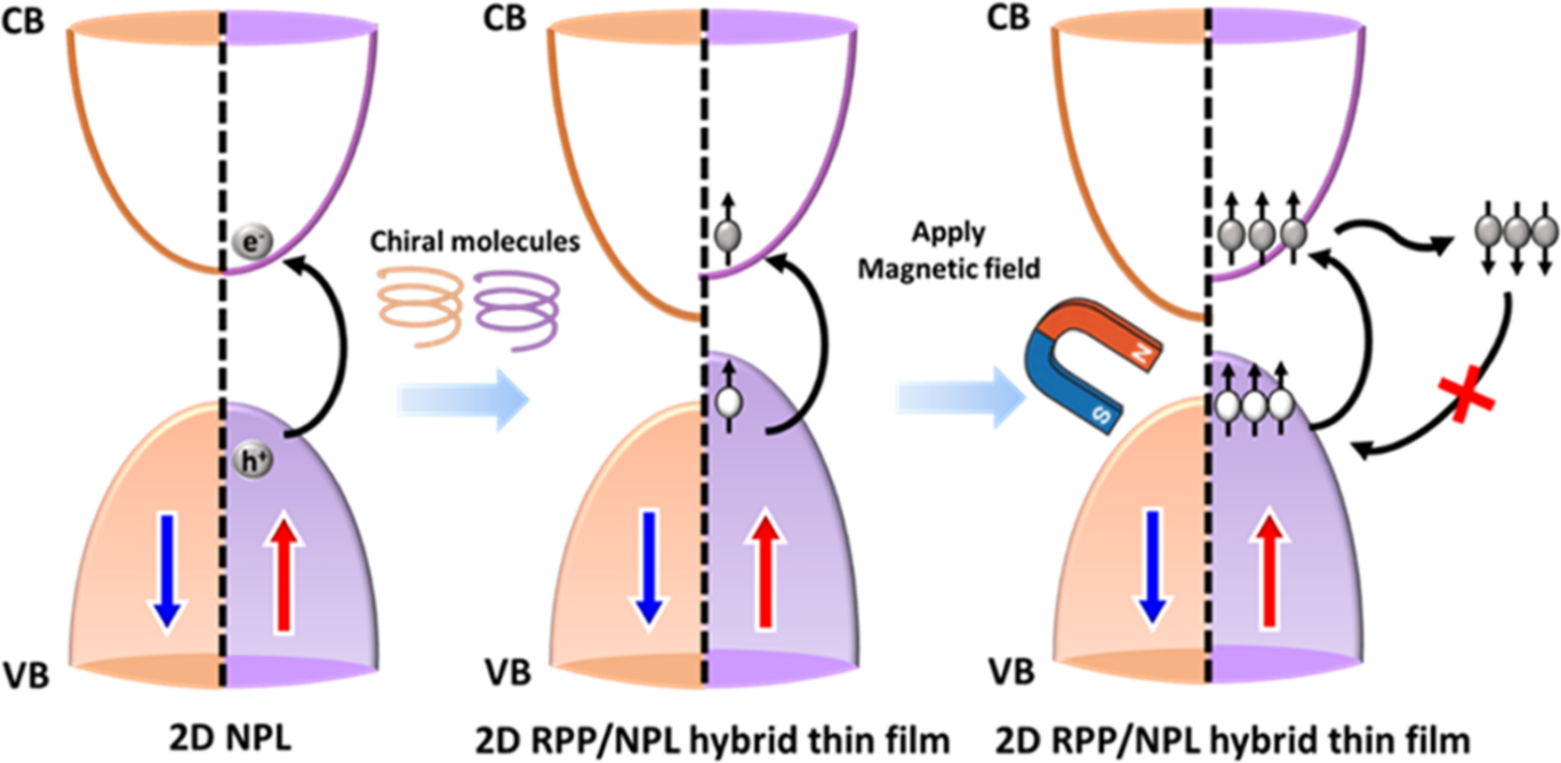
Schematic illustration of the prolonged photoexcited carrier lifetime induced by electron spin polarization under an external magnetic field in chiral 2D RPP/NPL.

## Conclusions

In conclusion, this work demonstrates the engineering of spin-polarized (R)- and (S)-2D RPP/NPL hybrid perovskite thin films by adding the chiral molecules (MBA/Br) into all-inorganic CsPbBr_3_ perovskite NPLs using a two-step synthesis approach. Within the 2D RPP/NPL hybrid structure, the chiral 2D RPP perovskites provide a substantial chiroptical response that facilitates the generation of spin-polarized electrons. The addition of chiral molecules significantly boosts the photocatalytic efficiencies of CO_2_ reduction in the 2D RPP/NPL hybrid perovskite thin films compared to pristine CsPbBr_3_ NPLs. This enhancement is primarily attributed to (1) the generation of chirality-induced spin-polarized electrons that exhibit longer carrier lifetimes and lower recombination rates under light illumination. (2) Furthermore, applying an external magnetic field induces Zeeman splitting in the spin-polarized bands of the chirality-regulated 2D RPP/NPL hybrid films, which further increases the populations of spin-polarized electrons and enhances photocatalytic CO_2_ reduction efficiencies. This work highlights chirality-induced spin polarization as an effective strategy to enhance photocatalytic performance, presenting a promising pathway for the development of efficient solar-to-fuel conversion systems. Despite these advances, several challenges remain. In particular, improving the long-term stability of hybrid films under continuous illumination and operational conditions is critical. Additionally, scalable synthesis methods that can reliably maintain chirality transfer and spin polarization have yet to be realized.

## Supplementary Material



## Notes

Synthesis of CsPbBr_3_ nanoplates and materials characterization (PDF)
