# Supplementary material for: Chirality-Regulated Spin-Polarization of Perovskite Nanoplates for Photocatalytic CO2 Reduction Reaction
Source: J Am Chem Soc. 2025 Aug 19;147(44):40347–55. doi: 10.1021/jacs.5c11357 (PMC12593411; doi:10.1021/jacs.5c11357)
Supplement: Supplementary file 1 [file ja5c11357_si_001.pdf]

Supporting Information

Chirality-regulated Spin-Polarization of Perovskite Nanoplates for Photocatalytic CO_2 Reduction Reaction

Cheng-Chieh Lin,[†,‡,#] Shao-Ku Huang,[▲,#] Wei-Ni Tseng,[±] Chun-Jen Su,[¶] Chao-Ching Huang,[±] Chih-Ying Huang,[†,‡] Cheng-Yu Yu,[▲] Man-Hong Lai,[ǁ] Jia-Yuan Sun,[&] Yu-Chiang Chao,[Δ] Hua-Shu Hsu,[§] Chih-Wei Luo,[&,◊,¶ǁ] Yu-Ming Chang,[ǁ] Chia-Chun Chen[±,*] and Chun-Wei Chen[†,▲,ǁ,□,*]

[†] International Graduate Program of Molecular Science and Technology (NTU-MST), National Taiwan University, Taipei 10617, Taiwan

[‡] Molecular Science and Technology Program, Taiwan International Graduate Program (TIGP), Academia Sinica, Taipei 11529, Taiwan

[▲] Department of Materials Science and Engineering, National Taiwan University, Taipei 10617, Taiwan

[±] Department of Chemistry, National Taiwan Normal University, Taipei 11677, Taiwan

[¶ǁ] National Synchrotron Radiation Research Center, Hsinchu 300092, Taiwan

[ǁ] Center for Condensed Matter Sciences, National Taiwan University, Taipei 10617, Taiwan

[&]Department of Electrophysics, National Yang Ming Chiao Tung University, Hsinchu 30010, Taiwan

[◊]Institute of Physics and Center for Emergent Functional Matter Science, National Yang Ming Chiao Tung University, Hsinchu 30010, Taiwan

[Δ]Department of Physics, National Taiwan Normal University, Taipei 11677, Taiwan

[§]Department of Applied Physics, National Pingtung University, Pingtung 90044, Taiwan

[□]Center of Atomic Initiative for New Materials (AI-MAT), National Taiwan University, Taipei 10617, Taiwan

[#] These authors contributed equally.

[*] Correspondence and requests for materials should be addressed to C.-C. Chen (email: cjchen@ntnu.edu.tw) and C.-W. Chen (email: chunwei@ntu.edu.tw).

1. Synthesis of $CsPbBr_3$ NPLs

The synthesis of cesium oleate (Cs-oleate) followed the procedure reported by Protesescu et al. [1]. In a 25 mL three-necked flask, 125 mg of cesium carbonate (Cs_2CO_3, 99%, Aldrich), 0.5 mL of oleic acid (OA, ~70%, Fisher), and 5 mL of 1-octadecene (ODE, 90%, Acros) were combined. The mixture was initially heated to 120 °C under vacuum for 1 hour to remove residual moisture and gases. Following this, the reaction temperature was raised to 150 °C and maintained for 20 minutes under an argon atmosphere. The resulting Cs-oleate solution was cooled and stored in an inert environment inside a glovebox for subsequent use.

The pristine three-dimensional (3D) all-inorganic perovskite $CsPbBr_3$ nanoplates (NPLs) were synthesized based on the method of Alivisatos et al. [2]. Lead(II) bromide ($PbBr_2$, 0.069 g, 99%, Aldrich) and ODE (5 mL) were loaded into a 25 mL three-necked flask and degassed under vacuum for 1 hour at 120 °C. Subsequently, dried oleylamine (OLA, 0.5 mL, 70%, Aldrich) and oleic acid (OA, 0.5 mL) were injected into the reaction mixture at 120 °C under an argon atmosphere. Once $PbBr_2$ was fully dissolved, the temperature was adjusted between 130 °C and 90 °C. A pre-prepared Cs-oleate solution (0.4 mL, 0.125 M in ODE, heated to ~100 °C) was then swiftly injected into the reaction mixture. To terminate the reaction and stabilize the colloidal solution, the system was rapidly cooled using an ice-water bath, yielding pristine $CsPbBr_3$ NPLs.

Here, we show the thickness of $CsPbBr_3$ perovskite NPLs measured by AFM, revealing a thickness of approximately ~3 nm. This observation is consistent with the image of high-resolution transmission electron microscopy (HR-TEM) as shown in **Figure 1(b)**.

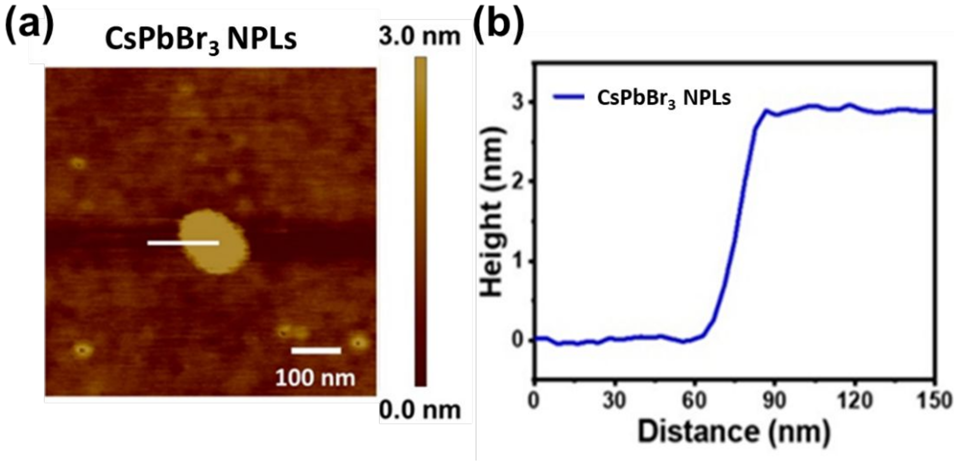

Figure S1. (a) The AFM images and (b) cross-sectional line profile exhibit the thickness of $CsPbBr_3$ NPLs.

2. Synthesis of (R-/S-) 2D RPP/NPL hybrid perovskite thin film by a

two-step process

First, a solution of (R-/S-) methylbenzylamine (10 mL, 78 mmol) was prepared in 30 mL of ethanol in an ice bath. To this solution, a concentrated aqueous solution of hydrobromic acid (HBr, 48%, 13.2 mL, 116 mmol) was added dropwise under vigorous stirring. Following the complete addition of HBr, the reaction mixture was allowed to remain in the ice bath overnight to facilitate crystallization. The resulting solid yellow powder of methylbenzylammonium bromide (MBA:Br) was isolated by rotary evaporation at 70 °C to remove residual organic solvents. The obtained solid was thoroughly washed with cold diethyl ether until the washings were colorless, and the product was subsequently dried under a vacuum to remove any remaining solvent. To enhance purity, the crude MBA:Br precipitate was recrystallized twice in isopropanol, yielding white colorless crystals.[3]

As shown in **Figure S2**, in the two-step synthesis protocol, chiral MBA:Br molecules are initially deposited onto a substrate via spin coating at 1000 rpm for 30 seconds while heating the substrate to 70 °C. This controlled heating facilitates the evaporation of residual solvents, resulting in the formation of uniform chiral molecular thin films. In the subsequent step, $CsPbBr_3$ perovskite NPLs are spin-coated onto the chiral molecular film under the same conditions (750 rpm, 30s). During this process, structural changes, including the formation of cracks and nucleation sites, occur due to the interaction between the deposited perovskite and the underlying chiral MBA:Br layer. These structural disruptions promote phase transformations during recrystallization. [4] The interplay between the structural cracking of the perovskite and the interaction with chiral MBA:Br molecules facilitates the generation of nucleation sites, leading to the formation of (R-/S-) 2D RPP/NPL hybrid perovskite thin films.

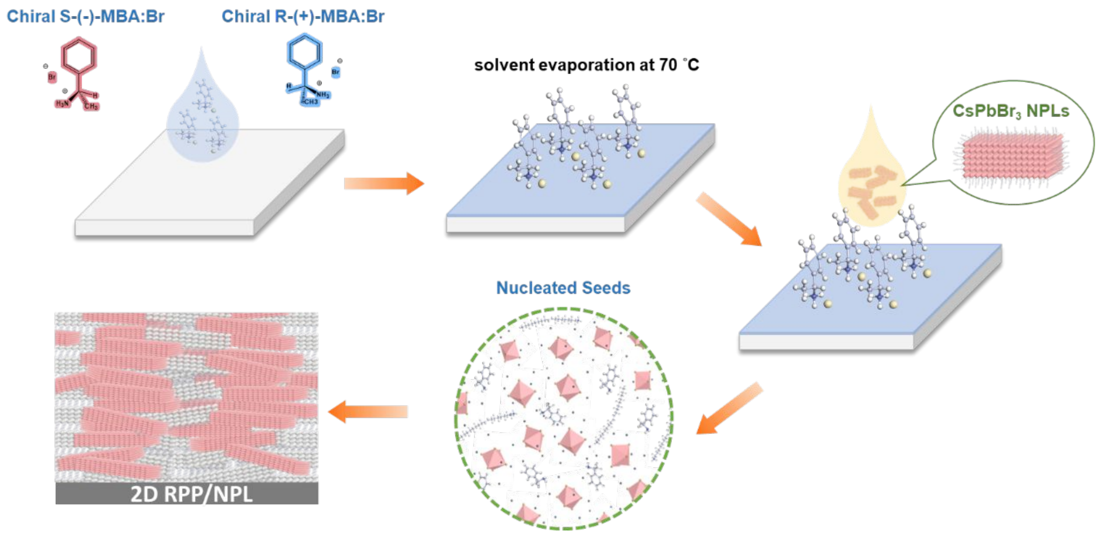

Figure S2. Schematic diagram of the two-step process for the synthesis of (R-/S-) 2D RPP/NPL hybrid perovskite thin film

We have provided X-ray diffraction (XRD) measurements, which clearly demonstrate the presence of diffraction peaks corresponding to 3D $CsPbBr_3$ perovskite phases in the 2D RPP/NPL hybrid samples.

As shown in **Figure S3**, both the (R)- and (S)-2D RPP/NPL hybrid samples exhibit prominent XRD peaks at $2\theta \approx 15.2°$, 21.4°, and 30.5°, which correspond to the (100), (101), and (200) planes of 3D $CsPbBr_3$, respectively. These peaks are also present in the pristine $CsPbBr_3$ NPLs, confirming the coexistence of the 3D perovskite phase in the hybrid films. The presence of these peaks supports the conclusion that parts of the film retain the 3D crystalline characteristics of $CsPbBr_3$.

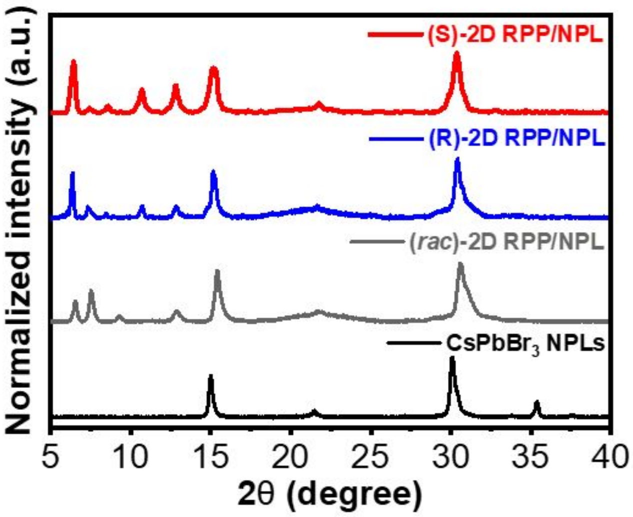

Figure S3. XRD patterns of $CsPbBr_3$ NPLs and 2D RPP/NPL hybrid films.

3. Materials characterization

The morphology of the $CsPbBr_3$ NPLs was investigated using a Philips Tecnai F30 field emission gun transmission electron microscope (FEG-TEM) operated at an accelerating voltage of 300 keV. High-resolution TEM (HRTEM) imaging was conducted utilizing the FEI Tecnai system for detailed structural analysis. In situ grazing-incidence wide-angle X-ray scattering (GIWAXS) measurements were performed at the 23A SWAXS beamline of the Taiwan Light Source (TLS) at the National Synchrotron Radiation Research Center (NSRRC), Hsinchu. An X-ray beam with an energy of 10.0 keV and an incident angle of 0.15° was employed for GIWAXS measurements. The scattered X-rays were collected using a Pilatus 1M-F area detector and a CMOS flat panel detector (C10158DK-3957(X)), positioned at a sample-to-detector distance of 176.50 mm. With the sample surface oriented in the xy-plane and the X-ray beam (wavelength λ) incident in the xz-plane, the scattering wavevector transfer q = (q_x,q_y,q_z) was defined as follows: $q_x = 2\pi\lambda^{-1}(cos\beta cos\phi - cos\alpha)$, $q_y = 2\pi\lambda^{-1}(cos\beta sin\phi)$, and $q_z = 2\pi\lambda^{-1}(sin\alpha + sin\beta)$, where α and β denote the

incident and exit angles, respectively, and ϕ represents the azimuthal angle deviating from the xz-plane. The in-plane q_r and out-of-plane q_z scattering vector components are given by: $q_r = (q_x^2 + q_y^2)^{1/2}$. [5] The scattering wavevector q of the GIWAXS measurements was calibrated using silver-behenate and LaB_6 reference powders. Photoluminescence (PL) and time-resolved photoluminescence (TRPL) spectroscopy were carried out using a Hitachi F-7000 spectrometer equipped with a 380 nm laser excitation source. UV-Vis absorption spectra were recorded with a Hewlett-Packard 8453 absorption spectrophotometer for optical characterization.

The circular dichroism (CD) and magnetic circular dichroism (MCD) measurements were performed using a GMW 3472 bipolar electromagnet with a Jasco J-815 CD spectrometer. Illumination was provided by a 450 W Xe lamp serving as the light source. The electromagnet was configured to generate an adjustable magnetic field ranging from 0 to 0.8 T, oriented perpendicular to the sample. The white light was directed through a monochromator to produce linearly polarized light, which was subsequently modulated by a photoelastic modulator to alternate between left- and right-handed circularly polarized light at a frequency of 50 kHz. The CD and MCD signals were acquired by measuring the differential absorbance of circularly polarized light under the influence of the applied magnetic field, using a photomultiplier tube as the detector. Circularly Polarized Luminescence (CPL) spectra were measured using a JASCO CPL-300 spectrophotometer, with an excitation wavelength of 350 nm and a detection range of 390–650 nm.

4. The control sample of rac-2D RPP/NPL

Racemic methylbenzylammonium bromide (rac-MBA:Br) was utilized to fabricate achiral perovskite films as control samples. The synthesis of rac-MBA:Br followed the

same procedure described for the preparation of (R-/S-) methylbenzylammonium bromide (MBA:Br). The rac-2D RPP/NPL hybrid films were fabricated using a two-step method. As depicted in **Figure S4(a) and (b)**, no discernible signals were observed in either the circular dichroism (CD) or circularly polarized luminescence (CPL) spectra for the rac-2D RPP/NPL hybrid perovskite films, indicating the absence of chirality in the control samples.

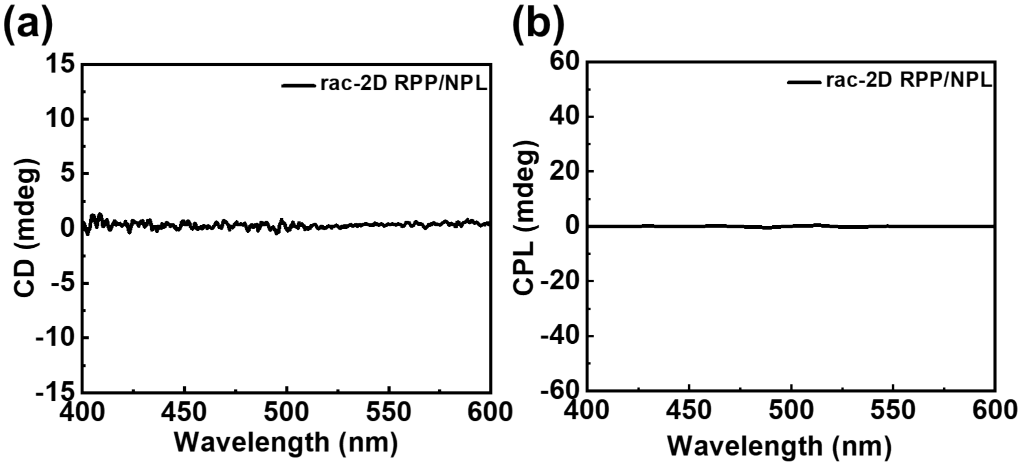

Figure S4. (a) The CD spectra of rac-2D RPP/NPL (b)The CPL spectra of rac-2D RPP/NPL

We next evaluated the photocatalytic CO_2 reduction reaction (CO_2RR) performance of rac-2D RPP/NPL by exposing the material to CO_2-saturated water vapor under simulated solar illumination (AM 1.5G, 100 mW cm^{-2}) for 6 hours. The rac-2D RPP/NPL exhibited a CO production yield of 15.3 μmol g^{-1}, which is comparable to that of pristine $CsPbBr_3$ NPLs (15.1 μmol g^{-1}), indicating a similar photocatalytic activity. In contrast, significantly higher CO yields were observed for the (R)- and (S)-2D RPP/NPL hybrid thin films, which reached 45.5 μmol g^{-1} and 30.6 μmol g^{-1}, respectively, as shown in **Figure S5(a)**. These results further underscore the role of chirality in enhancing photocatalytic CO_2RR performance.

When an external magnetic field of 0.3 T was applied to enhance the

photocatalytic performance of rac-2D RPP/NPL hybrids, the CO production yield remained nearly unchanged, increasing only slightly from 15.3 μmol g^{-1} at 0 T to 17.6 μmol g^{-1} at 0.3 T. In contrast, the (R)- and (S)-2D RPP/NPL hybrid thin films exhibited a substantial enhancement in CO production under the same magnetic field. By contrast, the (R)-2D RPP/NPL increased from 45.5 μmol g^{-1} at 0 T to 75.3 μmol g^{-1} at 0.3 T, while the (S)-2D RPP/NPL rose from 30.6 μmol g^{-1} to 49.3 μmol g^{-1} over a 6-hour period. These results indicate that the photocatalytic behavior of rac-2D RPP/NPL closely resembles that of pristine $CsPbBr_3$ NPLs, which also show no enhancement in response to an external magnetic field. (**Figure S5(b)**). In sharp contrast, the (R)- and (S)-2D RPP/NPL hybrids demonstrate approximately fivefold and threefold enhancements, respectively, relative to the pristine $CsPbBr_3$ NPLs and rac-2D RPP/NPL under a 0.3 T field. This pronounced difference highlights the critical role of chirality in modulating photocatalytic CO_2 reduction activity, both in the presence and absence of an external magnetic field.

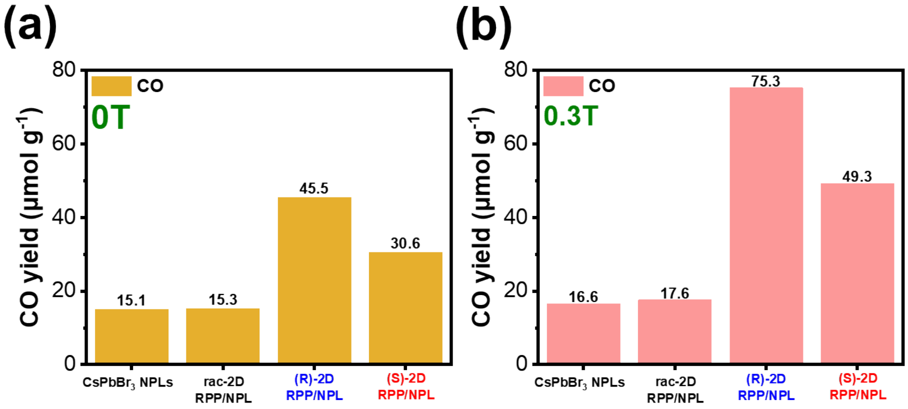

Figure S5. Photocatalytic CO yield measurements of $CsPbBr_3$ NPLs, rac-2D RPP/NPL, (R)-2D RPP/NPL, and (S)-2D RPP/NPL under (a) no magnetic field and (b) an external magnetic field (0.3 T) during 6 hours of visible-light irradiation.

5. Photocatalytic CO_2 reduction reaction (CO_2RR)

$CsPbBr_3$ NPL and (R-/S-) 2D RPP/NPL hybrid thin films were fabricated for application in photocatalytic CO_2 reduction reactions (CO_2RR). Initially, quartz substrates underwent a rigorous cleaning process using acetone, followed by heating to 70 ^oC. The $CsPbBr_3$ NPL was then deposited onto the preheated quartz substrates via a spin-coating technique at 750 rpm for 30 seconds. The coated substrates after the post-deposition were subjected to annealing at 80 ^oC under vacuum for 1 hour to enhance film stability and remove residual surface ligands.

5.1 Standard procedures for CO_2RR measurement

The samples were positioned within an aluminum reactor outfitted with quartz windows to facilitate photocatalytic CO_2 reduction reaction experiments. A schematic representation of the reaction system is provided in **Figure S6**. To prepare the reactor environment, residual air was removed through degassing, followed by purging with water vapor-saturated CO_2 for 30 minutes. The CO_2RR process was conducted under simulated solar illumination (AM 1.5G, 100 mW/cm^2) using a Newport LSH-7320 light source positioned above the reactor. Following the CO_2RR, the gaseous reaction products were collected and analyzed via gas chromatography-mass spectrometry (GC-MS) using a Shimadzu GCMS-QP2020 NX system equipped with a Restek CP-PoraBOND Q column and sample loop. The GC oven temperature was programmed as follows: an initial temperature of 25 ^oC (held for 1 min), ramped to 50 ^oC at 5 ^oC/min, and subsequently to 150 ^oC at 30 ^oC/min with a split ratio of 1:10 using helium as the carrier gas. Column flow mode was employed, with a carrier gas flow rate of 1.83 mL min^{-1}. The ion source was maintained at 200 ^oC. Data analysis, including peak area integration, was performed using Shimadzu GCMSsolution software. Calibration

curves for CO and CH_4, depicted in **Figures S7 (a) and (b)**, ensured precise quantification of product yields. For experiments involving an applied external magnetic field, permanent magnets were strategically placed beneath the aluminum reactor to augment spin polarization in the photogenerated charge carriers, further enhancing the photocatalytic activity.

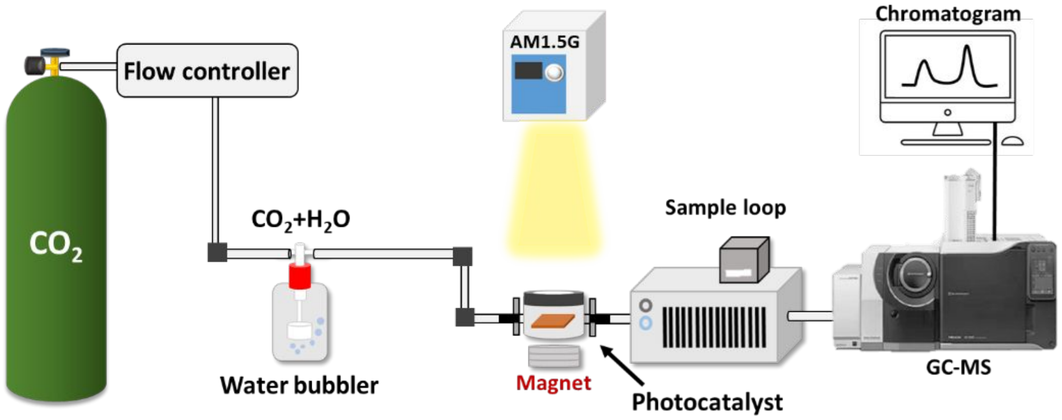

Figure S6. The schematic diagram of the setup of the system experiments for photocatalytic CO_2RR

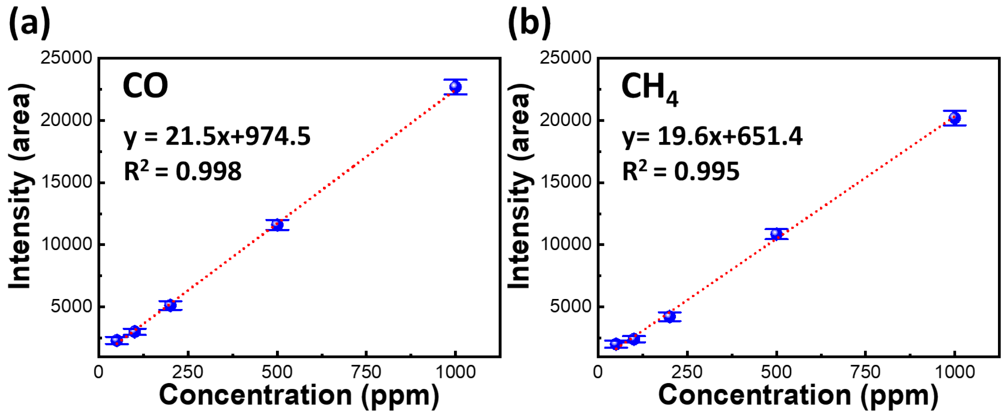

Figure S7. The calibration curves for (a) CO and (b) CH_4 were obtained using high-purity reference gases. The high linear correlation observed in the calibration data confirms the precision and reliability of the quantification methodology employed.

5.2 Photocatalytic CO_2RR performance under an external magnetic field

As illustrated in **Figure S8**, the photocatalytic generation rates of CO and CH_4 on $CsPbBr_3$ NPL and (R-/S-) 2D RPP/NPL hybrid thin films under varying external magnetic field (0 and 0.3 T) exhibited a continuous increase during 6 hours of irradiation. Photocatalytic product analysis was conducted at 2-hour intervals, demonstrating an approximately linear growth trend in the yields of CO and CH_4 over time. Specifically, the (R-/S-) 2D RPP/NPL hybrid thin films displayed a pronounced enhancement in the product yield rates of CO and CH_4 upon applying a 0.3 T external magnetic field. In contrast, the CO and CH_4 production rates of pristine $CsPbBr_3$ NPLs remained largely unaffected under identical magnetic field conditions. This marked disparity underscores the capability of an external magnetic field to significantly enhance the photocatalytic conversion efficiencies of (R-/S-) 2D RPP/NPL hybrid thin films. Furthermore, the application of a small, easily implementable permanent magnet (0.3 T) enables efficient performance enhancement, offering practical feasibility for photocatalytic processes.

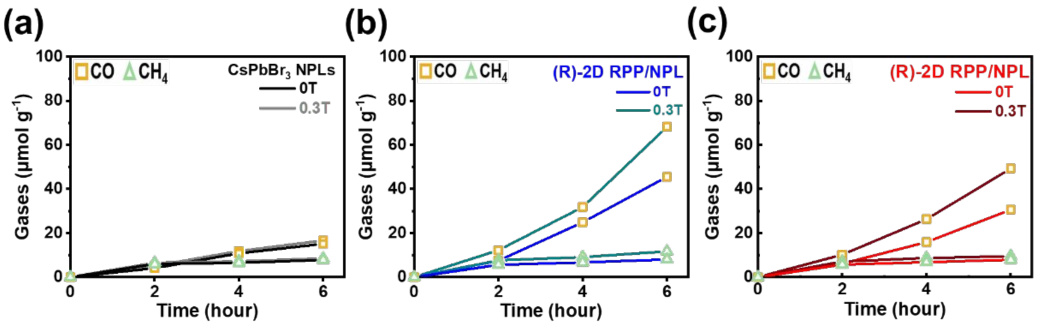

Figure S8. (a)$CsPbBr_3$ NPLs, (b)(R)-2D RPP/NPL, and (c)(S)-2D RPP/NPL with and without an external magnetic field (0 and 0.3T) during 6 hrs under AM1.5 irradiation.

5.3 Determining the origin of products in CO_2RR

To verify the origins of the photocatalytic products, CO and CH_4, during the photocatalytic reduction of CO_2 by $CsPbBr_3$ NPLs, two control experiments were

conducted over a 6-hour period without the application of an external magnetic field. In the first control experiment (Experiment 1), the CO_2 photocatalytic reaction was performed under illumination in an N_2-purged environment. In the second control experiment (Experiment 2), the reaction was carried out in a CO_2-saturated environment without illumination (dark conditions). As illustrated in **Figure S9**, these control experiments yielded only trace amounts of CO, markedly lower than the CO and CH_4 yields observed under standard experimental conditions (Experiment 3). This confirms that the long-chain surface ligands of $CsPbBr_3$ NPLs have minimal influence on the photocatalytic performance.

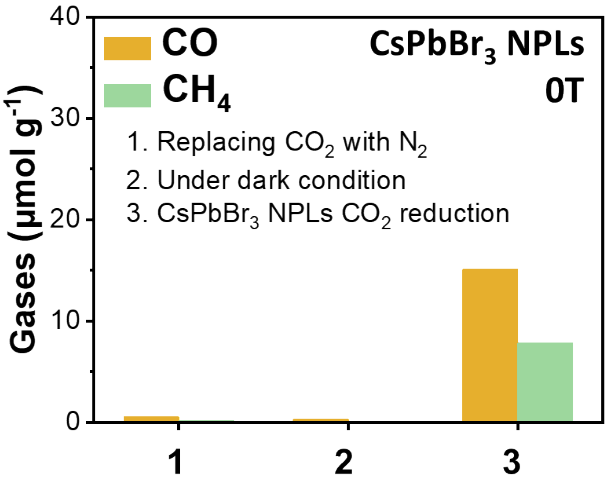

Figure S9. The photocatalytic CO_2RR product yields of $CsPbBr_3$ NPLs without an external magnetic field after a 6 h reduction reaction in different conditions.

Additionally, to address potential carbon contamination during the CO_2 photocatalytic process, isotope-tracing experiments were performed using isotopically labeled $^{13}CO_2$ gas (99%, $^{13}CO_2$, Cambridge Isotope Laboratories) by using gas chromatography-mass spectrometry (GC-MS). [6] As shown in **Figures S10(a) and (b)**, distinct signals corresponding to ^{13}CO (m/z: 29) and $^{13}CH_4$ (m/z: 17) were detected,

unequivocally confirming the reduction of CO_2 as the source of the photocatalytic products. The GC-MS analysis displayed well-resolved peaks with distinct retention times and corresponding mass spectra, clearly identifying CO and CH_4. The results were consistent with those of authentic reference standards, confirming the accurate identification of the target gases. The isotope-labeling experiments clearly demonstrate that both CO and CH_4 arise from the $^{13}CO_2$ feedstock, as confirmed by the expected mass shifts for ^{13}CO and $^{13}CH_4$ in the mass spectra. No other signal of unlabeled carbon species was detected, thereby indicating that no chiral molecules were decomposed during the photocatalytic reaction. These results demonstrate that the observed evolution of CO and CH_4 gases originates from the photocatalytic reduction of CO_2 rather than any contribution from the surface ligands of $CsPbBr_3$ NPLs.

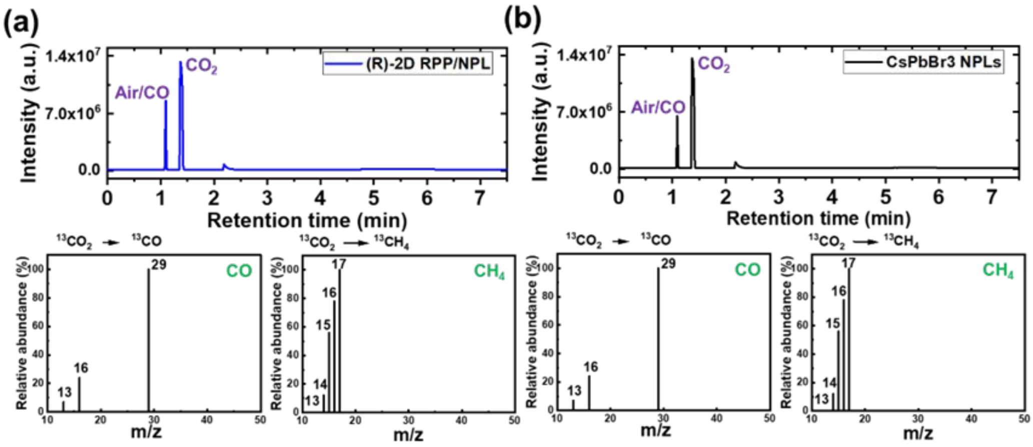

Figure S10. GC chromatograms and corresponding mass spectra of (a) R-2D RPP/NPL and (b) $CsPbBr_3$ NPL samples from the $^{13}CO_2$ isotopic reduction experiment. The mass spectra confirm the formation of ^{13}CO (m/z = 29) and $^{13}CH_4$ (m/z = 17), accompanied by clear GC signals for CO and CH_4.

6. Fourier Transform Infrared spectroscopy (FTIR)

To verify the successful incorporation of the chiral (R)-MBA:Br and (S)-MBA:Br

ligands used to modify the $CsPbBr_3$ NPLs, we conducted Fourier-transform infrared (FTIR) spectroscopy. As shown in **Figure S11**, the $CsPbBr_3$ NPLs modified with (R)-MBA:Br and (S)-MBA:Br exhibit distinct absorption bands in the 3100–3000 cm^{-1} region, corresponding to the symmetric and asymmetric stretching vibrations of sp^2-hybridized =C–H bonds on the aromatic ring. Additionally, characteristic bending vibrations (=C–H bending) of the aromatic ring are observed in the 1450–700 cm^{-1} region. These vibrational modes are typical spectral features of aromatic systems.

Monosubstituted benzene rings generally show pronounced aromatic C=C skeletal stretching in the 2000–1665 cm^{-1} range and out-of-plane C–H bending vibrations in the 750–690 cm^{-1} region. [7] In our study, these corresponding absorption features are clearly present in the spectra of the modified $CsPbBr_3$ NPLs, confirming the successful incorporation of the MBA aromatic structure. In contrast, the pristine $CsPbBr_3$ NPLs do not display these characteristic absorption bands, indicating the absence of aromatic functional groups and confirming that no chiral MBA:Br molecules are present on the unmodified sample surface.

Overall, the FTIR results provide direct and representative evidence that the chiral organic (R/S)-MBA: Br ligands have been successfully used to modify the $CsPbBr_3$ NPLs. The preservation of their characteristic aromatic functional groups further supports the presence of the chiral modifier within the perovskite nanostructure, strengthening confidence in the proposed modification mechanism.

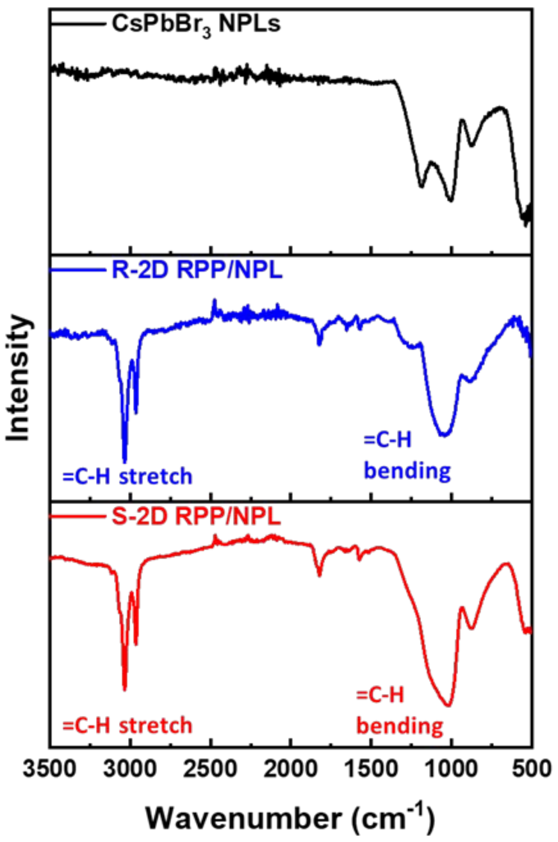

Figure S11. FTIR spectra comparing pristine $CsPbBr_3$ NPLs and ligand-modified $CsPbBr_3$ (R-/S-2D RPP/NPL hybrids)

7. Time-resolved photoluminescence (TRPL) measurement

We thank the reviewer for highlighting the need to clarify the fitting model and equation used to extract carrier lifetimes from the TRPL measurements. In our study, the time-resolved photoluminescence (TRPL) decay curves were analyzed using a biexponential decay function of the form:

$$I(t)=A_1 \exp(-t/\tau_1)+A_2 \exp(-t/\tau_2)$$

Where I(t) is the PL intensity at time t, A_1 and A_2 are the amplitudes, and τ_1 and τ_2 are the fast and slow decay time constants, respectively. This model accounts for both rapid surface recombination and slower bulk recombination processes, which are

typically observed in $CsPbBr_3$ NPLs, (R)-2D RPP/NPL, and (S)-2D RPP/NPL hybrid films.

The average carrier lifetime values reported in **Figure 4** were obtained using this model, ensuring consistency and comparability across all samples. The revised Supporting Information will include the full-fitting methodology, equations, and parameters for transparency and reproducibility.

Sample	A1	τ1(ns)	A2	τ2(ns)	τ_{avg}(ns)
CsPbBr3 NPLs (0T)	0.58	3.85	1.15	1.31	2.82
CsPbBr3 NPLs (0.3T)	0.58	3.89	1.14	1.31	2.85
(R)-2D/RPP/NPL (0T)	0.40	5.07	0.54	1.10	4.07
(R)-2D/RPP/NPL (0.3T)	0.45	7.01	0.49	1.35	6.02
(S)-2D/RPP/NPL (0T)	0.55	4.06	0.47	1.28	3.49
(S)-2D/RPP/NPL (0.3T)	0.47	5.41	0.45	1.35	4.61

Table S1. Summary of Time-Resolved Photoluminescence (TRPL) Carrier Lifetimes and Fitting Parameters for $CsPbBr_3$ NPLs, (R)-2D RPP/NPL, and (S)-2D RPP/NPL with and without an external magnetic field (0 and 0.3T).

References

(1) Protesescu, L.; Yakunin, S.; Bodnarchuk, M. I.; Krieg, F.; Caputo, R.; Hendon, C. H.; Yang, R. X.; Walsh, A.; Kovalenko, M. V. Nanocrystals of cesium lead halide perovskites ($CsPbX_3$, X= Cl, Br, and I): novel optoelectronic materials showing bright emission with wide color gamut. *Nano Lett.* **2015**, *15* (6), 3692-3696.

(2) Bekenstein, Y.; Koscher, B. A.; Eaton, S. W.; Yang, P.; Alivisatos, A. P. Highly luminescent colloidal nanoplates of perovskite cesium lead halide and their oriented assemblies. *J. Am. Chem. Soc.* **2015**, *137* (51), 16008-16011.

(3) Long, G.; Jiang, C.; Sabatini, R.; Yang, Z.; Wei, M.; Quan, L. N.; Liang, Q.; Rasmita, A.; Askerka, M.; Walters, G. Spin control in reduced-dimensional chiral perovskites.

Nat. Photonics **2018**, *12* (9), 528-533.

(4) Chen, Y.-C.; Wu, K.-C.; Chen, H.-A.; Chu, W.-H.; Gowdru, S. M.; Lin, J.-C.; Lin, B.-H.; Tang, M.-T.; Chang, C.-C.; Lai, Y.-H. Studies of high-membered two-dimensional Ruddlesden–Popper $Cs_7Pb_6I_{19}$ perovskite nanosheets via kinetically controlled reactions. *Mater. Horiz.* **2022**, *9* (9), 2433-2442.

(5) Baker, J. L.; Jimison, L. H.; Mannsfeld, S.; Volkman, S.; Yin, S.; Subramanian, V.; Salleo, A.; Alivisatos, A. P.; Toney, M. F. Quantification of thin film crystallographic orientation using X-ray diffraction with an area detector. *Langmuir* **2010**, *26* (11), 9146-9151.

(6) Wang, S.; Jiang, B.; Henzie, J.; Xu, F.; Liu, C.; Meng, X.; Zou, S.; Song, H.; Pan, Y.; Li, H. Designing reliable and accurate isotope-tracer experiments for CO_2 photoreduction. *Nat. Commun.* **2023**, *14* (1), 2534.

(7) Smith, B., Group wavenumbers and an introduction to the spectroscopy of benzene rings. **2016**.
